# Are volunteering and caregiving associated with suicide risk? A Census-based longitudinal study

**DOI:** 10.1186/s12888-019-2255-8

**Published:** 2019-10-11

**Authors:** Michael Rosato, Foteini Tseliou, David M. Wright, Aideen Maguire, Dermot O’Reilly

**Affiliations:** 1 Bamford Centre for Mental Health and Wellbeing, Ulster University, Cromore Road, Coleraine, BT52 1SA Londonderry, Northern Ireland; 2 Centre for Public Health, School of Medicine, Dentistry and Biomedical Science, Institute of Clinical, Sciences, Block B, Queen’s University Belfast, Belfast, BT12 6BA Northern Ireland

**Keywords:** Volunteering, Caregiving, Mental health, Suicide, Epidemiology, Mortality

## Abstract

**Background:**

Opposing risks have been identified between different prosocial activities, with volunteering having been linked to better mental health while caregiving has been associated with higher prevalence and incidence of depression. This study explored suicide risk of people engaged in prosocial activities of caregiving and/or volunteering.

**Methods:**

A Census-based record linkage study of 1,018,000 people aged 25–74 years (130,816 caregivers; 110,467 volunteers; and 42,099 engaged in both) was undertaken. Caregiving (light: 1–19; intense: ≥20 h/week), volunteering and mental health status were derived from 2011 Census records. Suicide risk (45 months follow-up) was assessed using Cox models adjusted for baseline mental health.

**Results:**

Intense caregiving was associated with worse mental health (OR_adj_ = 1.15: 95%CI = 1.12, 1.18) and volunteering with better mental health (OR = 0.87: 95%CI = 0.84, 0.89). For those engaged in both activities, likelihood of poor mental health was determined by caregiving level. There were 528 suicides during follow-up, with those engaged in both activities having the lowest risk of suicide (HR = 0.34: 95%CI = 0.14, 0.84). Engaging in either volunteering or caregiving was associated with lower suicide risk for those with good mental health at baseline (HR = 0.66: 95%CI = 0.49, 0.88) but not for their peers with baseline poor mental health (HR = 1.02: 95%CI = 0.69, 1.51).

**Conclusions:**

Although an increased risk of poor mental health was identified amongst caregivers, there was no evidence of an increased risk of suicide.

## Background

There is increasing societal interest in health outcomes, and especially the mental health, associated with prosocial activities such as volunteering and caregiving. However, although helping others is the common defining feature of these activities, there are significant differences between them both in terms of the characteristics of the participants and the attributes of the activities, such that they might be expected to have very different effects on mental health and suicide risk.

On one hand the ageing population, and a growing tendency to care for incapacitated people in their own homes, has placed an increasing reliance on ‘invisible health systems’ - the informal and unpaid help and assistance provided by family, friends and neighbours [1, 2]. A consensus is forming that caregiving is associated with poorer mental health: caregivers tend to demonstrate higher levels of stress [3–6] and a higher prevalence and incidence of depression, associations that may be more marked amongst people caring for those with dementia [7–12]. A dose-response relationship is evident with more intensive levels of caregiving associated with a higher likelihood of scoring above the clinical threshold for the Clinical Interview Schedule [13]. Caregivers may also be exposed to a wider range of stressors including exposure to domestic violence, financial difficulties or stressful life events and in some UK-based studies, were more than twice as likely to report suicidal thoughts and wishing they were dead in the previous week compared to non-carers [14]. Similar findings have been reported from Australia where O’Dwyer et al found that 7.1% of middle-aged female carers felt that life was not worth living and that this was very strongly related to the presence of depression [15]. Furthermore, 26% of caregivers looking after family members with dementia had contemplated suicide more than once in the previous year, and almost 30% said they were likely to attempt suicide in the future [16].

Volunteering, on the other hand, is increasingly advocated as a win-win activity that brings tangible benefit to the volunteer as well as the wider society. A recent meta-analysis [17] and narrative review [18] concluded that there is now considerable evidence that volunteering is associated with less depression, more positive affect and happiness, and greater life satisfaction, though it is also acknowledged that most of the included studies were cross-sectional. There remains however, some uncertainty as to whether the benefits of volunteering are limited to older people, as most of the subjects were older, or diminish at higher levels of volunteering commitment, possibly due to role exhaustion [19]. The likelihood of a causative protective relationship has been increased by a strong theoretical underpinning, initially encapsulated by Wilson and Musick [20] and later echoed by Li and Ferraro [21], which lists some of the bio-psycho-social mechanisms by which volunteering should lead to better mental health: it requires participation which provides a sense of purpose and value [22, 23]; it increases social networks and interactions, strengthening existing friendships and establishing new ones [24–27]; and finally, providing help to others can be a self-validating experience that enhances personal efficacy [28]. Another factor that connects volunteering and good mental health is the close correlation between volunteering and religiosity [29, 30] and one that differentiates it from caregiving is that it is more socially valued and publicly recognised [31]. These mechanisms may explain why volunteering is seen to act as a buffer against stress, work loss or bereavement [32–35].

Collectively these studies suggest that caregivers and volunteers should have different levels of mental ill-health and attendant suicide risk. However, many of the findings related to caregiving were based on relatively small studies or of select sub-populations that may not be representative of the wider community of caregivers. Furthermore, an increasing number of studies emphasise the many positive psychosocial aspects to caregiving [36, 37] and recent studies suggest that, despite the recognised stresses and other difficulties of caregiving, it may be associated with reduced rather than raised risks of all-cause mortality [38–43]. It is therefore possible that this relationship also holds for caregiving and suicide mortality.

This study aims to (i) compare the prevalence of poor mental health amongst volunteers and caregivers after adjustment for demographic and socio-economic factors; (ii) measure the risk of suicide amongst caregivers and volunteers, controlling for baseline health status and possible health selection effects - for example the degree of physical capability required to initiate and maintain a significant caregiving role [44]; and (iii) determine if these prosocial activities reduce suicide risk for those with poor mental health.

## Methods

The Northern Ireland Mortality Study (NIMS) is a record-linkage study comprising the Census returns for the whole enumerated population and subsequently registered deaths. Details of NIMS and how the linkage was conducted have been previously reported [45]. For this study, we defined the population-at-risk as those enumerated in the Northern Ireland Census (March 2011), aged 25–74 years and not living in institutional care, with mortality follow-up from the Census until December 2014 (45 months). All personal characteristics were drawn from the Census and selected on the basis of their known association with suicide risk, including age (in 10-year bands); gender; and marital status (married, never married, and – as a single group - those widowed, separated or divorced). Due to the ethnic homogeneity of Northern Ireland we summarised ethnicity as white and non-white. Regarding household variables, households were summarised as single person or not, on the basis of number of residents. Socioeconomic status was assessed using two parameters: (i) household car availability (two or more cars, one only, no household access), and (ii) a combination of housing tenure and capital value of the property. Data on housing tenure were drawn from the Census (grouped as owner occupiers, private renters or social renters), while capital value had been previously derived from a 2005 exercise by central government which aimed to determine the level of local payable tax of each household. These two variables were combined, producing an eight-fold classification of housing tenure/ property value: private renting; social renting; and, for owner-occupiers, five categories ranging from less than £75,000 to over £200,000 (Table 1), with owners of unvalued homes treated separately.

**Table 1:** Socio-demographic, socioeconomic and self-reported health characteristics by volunteering and caregiving status, for cohort members aged 25–74 years at the 2011 Census

	Non- caregiver and Volunteer	Caregiver only	Volunteer only	Caregiver and Volunteer
Proportion of Cohort (n)	734,618	130,816	110,467	42,099
Age (years)				
25–34	23.9	13.3	22.8	11.0
35–44	23.0	22.9	23.5	21.3
45–54	21.4	30.5	22.8	33.3
55–64	17.5	21.3	17.8	23.2
65–74	14.2	12.0	13.1	11.2
Sex				
Male	50.3	40.2	47.7	38.8
Female	49.7	59.8	52.3	61.2
Marital status				
Married	56.0	66.2	63.0	71.2
Never married	26.7	20.3	24.2	17.3
Widowed/Sep/Divorced	17.3	13.5	12.7	11.5
Single Person Household				
No	84.7	92.5	85.6	90.2
Yes	15.3	7.5	14.4	9.8
Religion				
Catholic	43.1	41.3	37.5	38.0
Presbyterian	21.3	22.2	22.3	23.1
Church of Ireland	15.4	16.3	14.3	14.7
Methodist	3.3	3.7	3.5	3.9
Other Christian	5.6	6.1	10.9	10.4
Other religions	0.9	0.8	1.3	1.1
No religion	10.4	9.7	10.2	8.8
Tenure/ property value				
£200 k	8.8	10.5	16.9	19.3
£150-199 k	11.3	13.1	16.8	19.1
£100-149 k	22.3	23.7	23.4	25.0
£75–99.9 k	14.2	14.4	10.9	11.0
< £75 k	9.7	9.0	6.0	5.8
Missing value	6.1	5.3	7.7	6.9
Private renting	1.6	1.3	1.8	1.3
Social renting	26.1	22.6	16.6	11.6
Household car access				
Two or more	46.7	48.4	59.9	63.5
One	37.7	39.5	31.9	31.3
None	15.6	12.1	8.2	5.2
Education				
Degree	28.6	27.5	52.3	50.2
Intermediate	40.0	44.0	37.7	40.7
No qualifications	31.4	28.5	10.0	9.2
Economic Activity				
Employed full-time	46.6	39.8	53.2	47.7
Employed part-time	15.5	18.6	19.0	22.8
Unemployed	4.5	3.4	3.9	3.2
Retired	16.2	16.1	15.2	15.7
Homemaker	3.9	12.3	2.8	5.8
Permanently sick	9.7	6.6	3.0	2.3
Other	3.6	3.3	2.9	2.4
Area				
Urban	37.7	40.2	37.0	35.9
Intermediate	34.9	32.9	32.0	30.4
Rural	27.5	26.8	31.0	33.7
Activity limitation				
No	74.9	76.9	85.3	83.3
A little	9.6	12.6	9.1	12.0
A lot	15.5	10.4	5.7	4.7
General health				
Very good	37.0	31.8	47.7	41.4
Good	35.9	42.0	38.0	42.7
Fair	18.3	20.9	11.9	14.3
Bad	6.9	4.4	2.0	1.4
Very bad	1.9	0.9	0.3	0.2
Chronic conditions				
Mental ill-health	9.0	8.4	5.0	5.7
Mobility problems	14.5	11.5	6.9	7.2
Chronic pain	14.1	14.0	8.4	10.2
Mortality				
Died by suicide	0.060	0.032		

### Caregiver and volunteer status

Census data were used to derive caregiving status from a caregiving-specific question: “*Do you look after, or give any help or support to family members, friends, neighbours or others because of either: long-term physical or mental ill-health/disability; problems related to old age?”* Four possible responses were available (none; caring for 1–19 h; 20–49 h; or 50 or more per week), with responders asked to disregard caring carried as part of paid employment. A volunteering-specific question was also asked: “*In the past year, have you helped with or carried out any voluntary work without pay?*” with responses limited to ‘*yes*’ / ‘*no*’.

### Assessment of chronic health status

The presence of chronic conditions which included mental ill health, chronic mobility problems and chronic pain was established through a unique item in the Northern Ireland Census asking about the presence of specific chronic conditions. The item was phrased as: “*Do you have any of the following conditions which have lasted, or are expected to last, at least 12 months*?”, with respondents ticking all items from a list that applied to them. For the purposes of the present study, chronic poor mental health was recorded when individuals reported they had: “*an emotional, psychological or mental health condition (such as depression or schizophrenia)*”; and chronic mobility problem or chronic pain if they said they reported: “*a mobility or dexterity difficulty (a condition that substantially limits one or more basic physical activities such as walking, climbing stairs lifting or carrying)”*, or … “*long-term pain or discomfort”.*

The linked data were anonymised and held in a safe setting by the Northern Ireland Statistics and Research Agency (NISRA). Access to the data was granted to the research team for this study. The use of the NIMS for research was approved by the Office for Research Ethics Committees Northern Ireland (ORECNI), while no formal consent was required.

### Outcome

The main outcome for analysis was risk of suicide during the follow-up period. In keeping with established practice both definite suicides and deaths of undetermined intent were combined to define suicide (ICD-10: X60-X84, Y10-Y34, Y87.0 and Y87.2). This reduced the possible effects of misclassification, though sensitivity analyses were undertaken using just definite suicides.

### Analysis strategy

This study recognises that many of those engaged in one type of prosocial activity may also engage in both - some researchers have called such people *super-helpers* [46, 47]. Therefore, while the correlates and outcomes of caregiving and volunteering are often reported separately, they are more usually described in a classification that recognises this overlap. The distribution of the dependant variable also determined the level of useful disaggregation of caregivers: for descriptive statistics and associations with poor mental health caregiving was classified as less or more intense – respectively, less than 20 h per week and 20 or more [48, 49]. However, because suicide is relatively rare, much of the mortality analysis used a classification that treated both volunteering and caregiving as binary measures.

Through the use of descriptive statistics at baseline, sociodemographic variations at baseline of volunteers and caregivers were examined. Furthermore, an investigation of the link between these activities and poor mental health was conducted using logistic regression models, adjusting for other demographic, socio-economic and physical health factors known to be associated with mental ill-health. However, economic activity was excluded - this includes a classification of persons unable to work because of long-term sickness/disability, a category known to include a significant proportion of persons reporting mental ill-health. Cox proportional hazards models examined the relationship between prosocial activity and suicide risk. Tests for interaction determined whether the mortality risk associated with volunteering and caregiving differed by age, sex or by baseline mental health status. Sensitivity analyses were carried out restricting suicide deaths to exclude deaths by undetermined intent and by excluding single person households as most caregiving is carried out between co-residents.

## Results

Of the 1,018,000 individuals included for analysis: 12.9% (130,816) were providing care and 10.9% (110,467) were volunteering. There was considerable overlap in these activities with 42,099 people engaged in both volunteering and caregiving, representing 32.2% of caregivers and 38.1% of volunteers.

Compared to those not engaged in any prosocial activity, non-volunteer caregivers were more likely to be older, female, married and slightly more affluent in terms of house value and car availability, though more likely to be in part-time employment and much more likely to be a homemaker. They were also less likely to report chronic mental health or mobility problems. Those involved in volunteering only were more similar to those not engaged in these prosocial activities in terms of age, sex, and household composition, though were more likely to belong to a more conservative Christian denomination. The socio-economic gradients are more evident amongst volunteers than caregivers and this is particularly marked for educational attainment. Volunteers had a lower prevalence of all three chronic health problems. Those engaged in both activities were more like caregivers in terms of age, sex, marital status and household composition but more similar to volunteers in terms of religious affiliation, socio-economic status and educational attainment.

### Prosocial activities and mental health

Table 2 shows the variation in likelihood of reporting poor mental health across all six categories of prosocial activity. The full models are available on request but show the usual socio-demographic and socioeconomic relationships to mental health including a higher prevalence in women (OR = 1.37: 95%CI = 1.34, 1.39) and people in single person households (OR = 1.33: 1.30, 1.36), marked gradients by educational attainment, housing tenure and property value and car availability. Those with chronic mobility difficulties or pain were more than twice as likely to report poor mental health (OR = 2.84: 95%CI = 2.78, 2.91 and OR = 2.52: 2.47, 2.58 respectively).

**Table 2:** Likelihood of having chronic mental health problems, by caregiver and volunteer status. Data represent Odds Ratios (and 95% Confidence Intervals) from separate logistic regression models

	M1: adjusted for age & sex	M2: M1 + demographic indicators^$^	M3: M2 + socio-economic indicators^$$^	M4: M3 + self-reported chronic mobility problems or pain
Non-Volunteer				
Non-Caregiver	1.00	1.00	1.00	1.00
Caring = 1–19 hours	0.58 (0.56,0.60)	0.65 (0.62,0.67)	0.81 (0.78,0.84)	0.92 (0.89, 0.95)
Caring = 20+ hours	1.11 (1.08,1.14)	1.30 (1.26,1.33)	1.07 (1.04,1.11)	1.15 (1.12, 1.18)
Volunteer				
Non-Caregiver	0.52 (0.50,0.53)	0.55 (0.53,0.57)	0.78 (0.75,0.81)	0.87 (0.84, 0.89)
Caring = 1–19 hours	0.45 (0.43,0.47)	0.52 (0.49,0.54)	0.83 (0.79,0.88)	0.96 (0.90, 1.01)
Caring = 20+ hours	0.71 (0.66,0.76)	0.85 (0.79,0.91)	1.07 (1.00,1.15)	1.17 (1.08, 1.25)

The likelihood of reporting poor mental health amongst caregivers varied by caregiving intensity: those providing less intense caregiving were marginally less likely to report poor mental health than those who undertook no prosocial activities while the more intense caregivers had worse mental health (OR = 1.15: 95%CI = 1.12, 1.18). Those undertaking only volunteering had the best mental health (OR = 0.87: 95%CI = 0.84, 0.89), though when this was combined with caregiving responsibilities the likelihood of poor mental health was more closely aligned to the level of caregiving.

### Suicide risk

Over the 45 months of follow-up there were 17,708 deaths to the cohort of which 528 were classified as suicide: 390 (73.9%) to men and 53.4% to people aged 35–54 years. Overall, 90 (17.0%) of suicide deaths were amongst the 27.8% of people engaged in prosocial activities: a more disaggregated breakdown was not possible due to disclosure rules associated with use of the data. Table 3 shows suicide risks by level of prosocial activity, drawn from models fully adjusted for socio-demographic, socio-economic and health factors. As with Table 2 the full models are not shown for brevity but are available on request. The omitted results confirm established suicide-related epidemiological socio-demographic and health profiles: for example, males more likely than females; those living alone more likely when compared to those in households of two people or more; and those reporting poor baseline mental health more likely than their peers reporting no mental health problems.

**Table 3:** Risk of mortality by suicide according to caregiver and volunteer status. Data represent Hazard Ratios (and 95% Confidence Intervals) from separate Cox proportional hazard models

Caregiver and volunteer status	M1 Adjusted for age and sex	M2 M1 + religion and marital status	M3 M2 + Single person household	M4 M3 + Socio-economic status	M5 M4 + Economic Activity	M6 M5 + Self-reported health status
Non-helper	1.00	1.00	1.00	1.00	1.00	1.00
Caregiver only	0.64 (0.47, 0.87)	0.69 (0.51, 0.94)	0.75 (0.55, 1.02)	0.76 (0.56, 1.03)	0.81 (0.59, 1.10)	0.80 (0.59, 1.09)
Volunteer only	0.60 (0.43, 0.83)	0.65 (0.47, 0.91)	0.64 (0.46, 0.89)	0.79 (0.56, 1.10)	0.84 (0.60, 1.17)	0.86 (0.62, 1.20)
Both Caregiver & Volunteer	0.22 (0.09, 0.53)	0.25 (0.10, 0.60)	0.25 (0.10, 0.61)	0.33 (0.13, 0.79)	0.35 (0.15, 0.86)	0.34 (0.14, 0.84)

The presented results show that in age and sex adjusted models cohort members providing either only caregiving or only volunteering had approximately one third lower risk of suicide than those who engaged in neither activity. A proportion of the lower risk for caregivers is due to differences in demographic factors such as being married or not residing in a single person household, as there was little further attenuation with addition of socio-economic or health factors. On the other hand, much of the lower suicide risk amongst volunteers disappeared with adjustment for socio-economic and health factors. Although the confidence intervals for volunteering only and caregiving were not significant, in a separate analysis that aggregated these prosocial activities, being engaged in either volunteering or caregiving was associated with a lower risk of death (HR = 0.77: 95%CI = 0.61, 0.97). Those cohort members who engaged in both caregiving and volunteering had a risk of suicide about one third of those who engaged in neither (HR = 0.34: 95%CI = 0.14, 0.84).

**Fig. 1:**
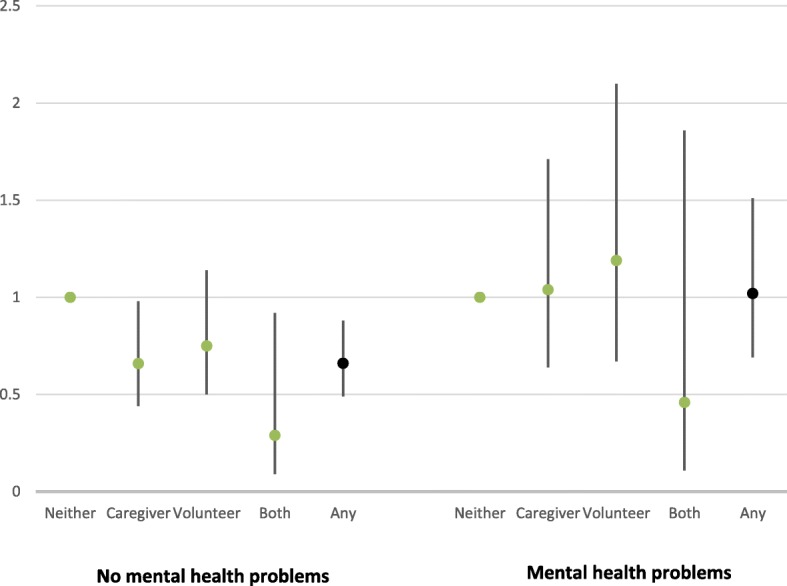
Risk of death due to suicide according to prosocial activity (neither activity, caregiver only, volunteer only, both caregiver and volunteer; any prosocial activity), stratified by presence of a chronic mental health condition. Data represents Hazard Ratios (and 95%CIs) from Cox Proportional Hazards models fully adjusted for all the variables listed in Table 1

Further analysis showed that the relationship between helping and suicide risk varied according to the presence of baseline poor mental health but not chronic mobility problems (*P* = 0.003 and *P* = 0.920 respectively). The results of the analyses stratified according to chronic poor mental health are shown graphically in Fig. 1; no mental health problems describing a *direct effect* of prosocial activity on suicide, while the presence of mental health problems presents an *indirect effect* through mental ill-health. Volunteers and caregivers with better mental health tend to have a lower risk of suicide but those with poor mental health tend to exhibit the same risk as those not engaging in either activity. This is confirmed in an analysis that considered all the helping activities together; for those with better mental health, prosocial activity is associated with a lower risk of suicide (HR = 0.66; 95%CIs 0.49, 0.88), for those with poor mental health the addition of helping activity does not improve models predicting suicide risk (*P* = 0.993) and the risk of suicide amongst helpers was the same as for non-helpers (HR = 1.02: 95%CI = 0.69, 1.51). Therefore, there is a decreasing *direct effect* of prosocial activity on suicide, though an opposing *indirect* effect was also observed in the presence of mental ill-health (as supported by the significant interaction coefficient), thus suggesting a complex link between prosocial activity, mental health and suicide risk.

### Sensitivity analyses

Two sets of sensitivity analyses tested the robustness of the findings. The first showed that the reduced risk associated with prosocial activity was not confounded by the definition of suicide used: restricting deaths to definite suicides (excluding ICD-10 codes Y10-Y34, where intent was not determined) did not change the lower risk associated with caring (HR = 0.76: 95%CI = 0.59, 0.99). Furthermore, as most caregiving is presumed to be to a co-resident we repeated the analyses excluding single person households rather than adjusting for household size, but again there was little change in the lower suicide risk associated with prosocial activity (HR = 0.77: 95%CI = 0.58, 1.01).

## Discussion

This large representative study shows that prevalence of mental ill-health is related to the type of prosocial activity – while more intensive caregivers record worse mental health than non-caregivers, volunteers record better mental health. Additionally, when compared to those who don’t engage in either prosocial activity, both caregivers and (separately) volunteers have reduced suicide risks, though in fully-adjusted models these associations lose significance. However, volunteers with caregiving responsibility maintain a significantly reduced suicide risk even after full adjustment. In the analysis of suicide stratified by absence/presence of mental health problems at baseline, those caregivers and volunteers with no mental health problems both recorded lower likelihoods of suicide mortality than their non-prosocial peers. However, for those with mental health problems a similar analysis showed no suicide mortality differences when compared with their non-prosocial peers. Thus, while volunteering is associated with lower likelihood of poor mental health [17, 18], it does not appear to be associated with reduced suicide risk for those with poor mental health. This is perhaps not surprising given the strength of the relationship between poor mental health and suicide [50, 51].

To our knowledge this is the first study to examine suicide risk amongst volunteers and caregivers and these findings provide some reassurance that despite the higher prevalence and incidence of depression and common mental disorders amongst caregivers [7–13] this does not translate into higher suicide risk. The majority of caregivers do not have poor mental health and their suicide risk is about one-third that of non-caregivers, and amongst the minority with poor mental health there is no evidence of an increased suicide risk. This may be surprising given reports suggesting increased suicide ideation [14–16] though, as Gunnell et al [52] point out, the epidemiology of suicide ideation and suicide completion is different and, importantly, the relationship can be modified, most obviously by age and sex. It appears that while a disproportionate number of caregivers suffer from increased suicide ideation, this does not generally translate into higher suicide risk. It may be that the benefits of caregiving outweigh the more negative aspects of the role. Altruistic behaviour has also been suggested to improve wellbeing and reduce mortality [39], while the increased sense of purpose which has been associated with caregiving has been known to act as a buffer against mortality risk across different adult age groups [53, 54], irrespective of other markers of psychological or affective well-being [55]). Although the psychological burden may be high, this sense of purpose may protect against completed suicide due to a sense of responsibility on the part of the caregiver.

This study also found a significant overlap in prosocial activities with approximately one quarter of volunteers and caregivers engaged in both activities and we would agree with other researchers that future studies should consider them simultaneously [56, 57]. Indeed, as there is some indication that people combining caregiving and volunteering have the lowest risk this suggests a synergism of actions and mechanisms [58], perhaps the increased sense of bonding associated with caregiving complements the outward-looking social engagement associated with volunteering. However, we are also aware that too many roles can offset the benefits and may result in worse health outcomes [19, 59].

There is a consensus that caring is associated with an increased risk of stress and poorer mental health. However, as researchers such as Brown [38] point out, the interpretation of this association is less clear and may be a consequence of witnessing the suffering or deterioration of a spouse or close family member with a chronic disabling illness, or of anticipatory bereavement rather than the effects of caring per se. Others, such as Roth [60], have pointed to results from national surveys showing high proportion of carers reporting satisfaction with their role and how this might be linked to forging stronger bonds with the care recipient [30–32]. However, some caring could be associated with stress and poor mental health and this is most likely when demands exceed available psychological, social or financial resources [34]. Personality traits might also be linked to caregiver burden, with neuroticism being negatively associated with mental health-related quality of life while extraversion has an opposite effect [61]. Volunteering has also been strongly related with extraversion [62], suggesting that personality traits could affect the relationship between prosocial activities and suicide risk. It is of note that a relatively recent twin study [63] has suggested that the interplay between caring and distress might also be due to a vulnerability which is shaped, at least in part, by genetic and early life factors.

This study has significant strengths and limitations that should be acknowledged. To our knowledge it is one of the largest and most representative studies of suicide risk for caregivers and volunteers. Caregiving and volunteering status were defined at baseline and the linked administrative data ensured accurate assessment of cause of death extracted from Official Statistics. However, we acknowledge that the data relating to prosocial activity is limited: there is no indication of the relationship between carer and the cared-for person or of the type of care provided. Earlier mortality studies have emphasised the role of carer stress, but here only the number of hours spent caring and baseline mental and emotional health status were available as a proxy for stress. The classification of volunteer status included no indication of its duration, nature or intensity - limiting our understanding of its interrelationship with more intense levels of caregiving. Furthermore, it was not possible to differentiate between volunteering undertaken as part of an organisation or in a private capacity. However, this seemingly naive instrument does identify the same demographic, socio-economic features and health and mortality relationships that characterise volunteers in other studies. Although the protective effect of engaging in both caregiving and volunteering activities in relation to suicide risk was highlighted, future researchers might wish to explore the potential influence of competing mortality risk on these effects. Additionally, despite the fact that this observational study shows that caregiving is associated with a reduced risk of suicide, it cannot definitively prove that caregiving reduces suicide risk. While a concern (not easily dismissed) is that caregivers and volunteers are healthier than those who are neither [44], after adjustment for health status, there is very little change in hazard ratios, suggestive that health selection was not a major contributor to suicide risk, even among those with poor mental health. Finally, it is worth considering that our sample is of predominantly white ethnicity, with variation in the associations of interest to be expected in people from other cultures: for example, in Asian countries where government support for informal carers is limited [64, 65] one study found that informal caregiving does not appear to be linked with all-cause mortality [66]; though this may not pertain when focusing on suicide risk.

One major point raised by this study relates to the additional evidence it provides that current putative health consequences associated with informal caregiving are too pessimistic. Two recent overviews, the first by Brown and Brown [67] the second by Roth et al [60] have argued for a more balanced perspective and state that ‘*Policy reports, media portrayals, and many research reports commonly present an overly dire picture of the health risks associated with caring and largely ignore alternative positive findings*’. There are growing concerns that this negative view of caregiving, with an over-emphasis on burden and stress, may deter some from undertaking a caregiving role. Therefore, while it will be increasingly important to identify how best to assess the physical and mental effects of the caring role so as to provide the most appropriate support [13, 14], it is important to recognise both the benefits accruing from caregiving and reiterate the more general observation that caregivers as a general group have reduced mortality risk compared to their non-caregiving peers.

## Conclusion

These analyses suggest benefits associated with engagement in pro-social activity (*caregiving* and/or *volunteering*): for *caregivers* the evidence proposes that, while there were increased risks of reporting mental ill-health, there was no evidence of increased suicide risk; for *volunteers* the risks of both reporting mental ill-health and suicide were reduced, though not significantly so in the more fully adjusted mortality models; and for persons engaging in both suicide risk was significantly reduced. However, while we stress these findings be approached with caution, it is important to further research the relationship between pro-social activity (especially *caregiving*) and health outcomes – if only because its societal role is predicted to grow.

## Data Availability

The data that support the findings of this study are available from the Northern Ireland Mortality Study (NIMS) but restrictions apply to the availability of these data, which were used under permission for the current study, and so are not publicly available. Access to the anonymised dataset can be obtained through an application process for data permission NILS Research Approvals Group (NILS-RAG).

## Abbreviations

95%CI: 95% Confidence Intervals
HR: Hazards Ratio
ICD-10: International Classification of Diseases-10th Revision
NIMS: Northern Ireland Mortality Study
NISRA: Northern Ireland Statistics and Research Agency
OR: Odds Ratio
ORECNI: Office for Research Ethics Committees Northern Ireland
